# Rapid Detection of *Brucella* spp. and Elimination of Carryover Using Multiple Cross Displacement Amplification Coupled With Nanoparticles-Based Lateral Flow Biosensor

**DOI:** 10.3389/fcimb.2019.00078

**Published:** 2019-03-28

**Authors:** Shijun Li, Ying Liu, Yue Wang, Ming Wang, Chunting Liu, Yi Wang

**Affiliations:** ^1^Laboratory of Bacterial Infectious Disease of Experimental Center, Guizhou Provincial Center for Disease Control and Prevention, Guiyang, China; ^2^Key Laboratory of Major Diseases in Children, Ministry of Education, National Key Discipline of Pediatrics (Capital Medial University), National Clinical Research Center for Respiratory Diseases, Beijing Key Laboratory of Pediatric Respiratory Infection Disease, Beijing Pediatric Research Institute, Beijing Children's Hospital, Capital Medical University, Beijing, China

**Keywords:** brucellosis, isothermal amplification technique, nano-biosensor, rapid diagnosis, limit of detection

## Abstract

*Brucella spp*.is capable of causing disease in a range of animal hosts, and human brucellosis is regarded as a life-threating disease. A novel isothermal amplification technique, termed multiple cross displacement amplification (MCDA), was employed for detecting all *Brucella* species strains. *Brucella-*MCDA targets the *Bscp31* gene (*Brucella* species-specific gene) to specifically design a set of 10 primers. The *Brucella-*MCDA can be coupled with nanoparticles-based lateral flow biosensor (LFB) for highly specific, simple, rapid, and visual detection of *Brucella*-specific amplicons. Using the protocol, a MCDA amplification followed by 2 min LFB resulted in visualization of DNA products trapped at the LFB test line. Various species of Gram-positive and Gram-negative strains are applied for optimizing and evaluating the target assay. Optimal MCDA condition is found to be 63°C for 40 min, with detection limits at 10 fg of templates in the pure cultures. The specificity of MCDA-LFB technique is of 100%, and no cross-reactions to non-*Brucella* strains are observed according to the specificity examination. Furthermore, dUTP and AUDG enzyme are added into the MCDA reaction mixtures, which are used for removing false-positive amplification generating from carryover contamination. Thus, 20 min for rapid template extraction followed by AUDG digestion (5 min), MCDA (40 min) combined with LFB detection (2 min) resulted in a total assay time of ~70 min. In sum, *Brucella*-MCDA-LFB technique is a rapid, simple, reliable, and sensitive method to detect all *Brucella* species strains, and can be used as potential screening tool for *Brucella* strains in various laboratories.

## Introduction

*Brucella spp*. are facultative intracellular bacteria that consists of six classical species: *Brucella melitensis* (*B. melitensis*), *B. abortus, B. canis, B. ovis, B. suis, B. neotomae*, one human origin species *B. innoponita*, and two marine species: *B. ceti* and *B. pennipedilis* (De et al., 2008). *Brucella* species are responsible for brucellosis in a range of animal hosts, including marine mammals, wildlife, domesticated livestock, and humans (Tiller et al., 2010). These bacteria are typically transmitted to humans through exposure to tissues or fluids from infected animals, or consumption of unpasteurized dairy products (Traxler et al., 2013). Brucellosis is the commonest zoonotic disease worldwide, which is characterized by fetal death and spontaneous abortion in animals, and chronic disease, focal complications, and severe flu-like symptoms in humans (de Glanville et al., 2017). Thus, *Brucella* spp. can cause economic losses to the livestock industry, and serious health problems to animals and humans. To effectively control and prevent these pathogens, the suitable diagnostic techniques using a sensitive, reliable, and rapid assay are required in field, clinical, and veterinary laboratories.

Diagnostic assays, including microbiological isolation of organisms, bacterial identification, and nucleic acid amplification-based methods, have been developed and used for detection of *Brucella* pathogens in various samples (Kattar et al., 2007). Although traditional bacteriological techniques of diagnosing *Brucella* are the gold standard, the method is time-consuming, and requires highly skilled technical personnel for handling live culture (Sagi et al., 2017). Serological tests, including Rose Bengal Plate Test and Standard Tube Agglutination Test, are not highly specific, and antibodies may cross react with other pathogens, such as *Francisella tularensis, E. coli* O:116, *Yersinia enterocolitica* serotype O:9, *Salmonella*, and *Pasteurella multocida* (Bounaadja et al., 2009). In addition, Brucella is a Select Agent in the USA and is also highly transmissible such that laboratorians frequently get infected when manipulating cultures. Molecular methods, such as polymerase chain reaction (PCR)-based techniques (conventional PCR, multiplex PCR and real-time PCR), are useful for detection of *Brucella* pathogens and diagnosis of brucellosis because these techniques are rapid, sensitive and specific (Kattar et al., 2007; Bounaadja et al., 2009; Kaden et al., 2017). However, PCR-based assays require costly specialized apparatus and skilled personnel, which limit their wider application in various fields, such as point-of-care testing, field diagnosis and more (Law et al., 2014). Herein, advanced techniques are required for simple, reliable and sensitive detection of target pathogens to ensure optimal therapy and management of patients.

The recently established isothermal amplification techniques are the next generation molecular tools, which due to their ruggedness, simplicity, and low cost could offer major advantages (Li and Macdonald, 2015). Although several isothermal amplification-based techniques, including loop-mediated isothermal amplification and recombinase polymerase amplification, have been reported to detection of *Brucella* pathogens, these assays been restricted due to the need for expensive laboratory instrument (real-time turbidimeter or fluorescence apparatus), expensive regents (calcein), and additional analysis procedure (agarose gel electrophoresis) to indicate assay's results (Song et al., 2012; Soleimani et al., 2013; Ren et al., 2016). Thus, a valuable diagnostic tool that is faster, simpler, less hazardous, and usually more sensitive is required for *Brucella* detection.

To achieve such effective detection technique, a novel isothermal amplification, termed multiple cross displacement amplification (MCDA), is employed to detect *Brucella* (Wang et al., 2015). MCDA technique is capable of amplifying nucleic acid sequences with high sensitivity and efficiency using simple reaction equipment at a fixed temperature. In the reaction system, a total of 10 primers (displacement primers F1 and F, cross primers CP1 and CP2, amplification primers C1, D1, R1, C2, D2, and R2) were added into reaction mixtures, which were specially designed on the target sequences, possessing high specificity for nucleic acid detection (Wang et al., 2015). In order to achieve faster analysis and simplify diagnostic tools, the standard MCDA method coupled with nanoparticle-based lateral flow biosensor (MCDA-LFB) has been reported at the recent publications, providing rapid, reliable, and visual detection of target pathogens in clinical diagnostics and serving as a point-of-care device (Wang et al., 2017, 2018a). More recently, MCDA-LFB method is integrated with AUDG (antarctic thermal sensitive uracil-DNA-glycosylase) cleavage to eliminate false-positive results, which are generated from carryover contamination (Wang et al., 2017, 2018a,b). As a potentially valuable detection technique for the reliable diagnosis of pathogen infection, the study reports on an assay for detection of *Brucella* spp. by MCDA-LFB method, and examines the assay performance with pure culture and clinical samples.

## Materials and Methods

### Reagents and Apparatus

Isothermal amplification kits, visual detection reagent (Malachite Green, MG) and dUTP were purchased from Bei-Jing HaiTaiZhengYuan. Co., Ltd. (Beijing, China). The backing card, sample pad, conjugate pad, nitrocellulose membrane (NC), and absorbent pad were purchased from the Jie-Yi Biotechnology. Co., Ltd. (Shanghai, China). Biotinylated bovine serum albumin (biotin-BSA) and rabbit anti-fluorescein antibody (anti-FITC) were obtained from Abcam. Co., Ltd. (Shanghai, China). Dye (Crimson red) streptavidin coated polymer nanoparticles (129 nm, 10 mg mL^−1^, 100 mM borate, pH 8.5 with 0.1% BSA, 0.05% Tween 20, and 10 mM EDTA) were purchased from Bangs Laboratories, INC. (Indiana, USA). Antarctic thermal sensitive uracil-DNA-glycosylase (AUDG), dATP, dTTP, dCTP, and dGTP was purchased from New England Biolabs, INC. (Beijing, China). The DNA extraction kits (QIAamp DNA minikits; Qiagen, Hilden, Germany) were obtained from Qiagen (Beijing, China).

### Primer Design

**Figure 1:**
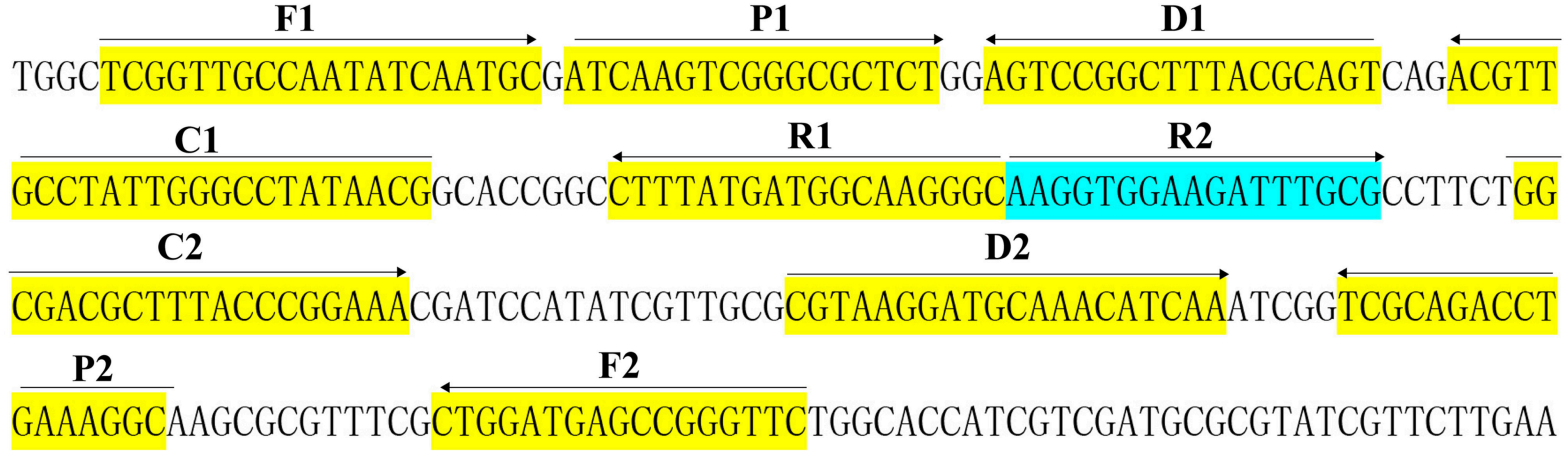
Sequence and location of *Bscp31* gene used to design multiple cross displacement amplification primers. The nucleotide sequences of the sense strand of *Bscp31* are listed. Right arrows and left arrows indicate sense and complementary sequences that are used.

A set of 10 primers targeted toward the *Bscp31* gene (Genbank accession no. M20404) of the *Brucella* spp. was designed using PRIMER PREMIER 5.0 software based on the conserved sequences examined by the alignment of the *Bscp31* gene sequences obtained from GenBank (https://blast.ncbi.nlm.nih.gov/Blast.cgi). Particularly, the *Bscp31* gene encodes a 31-kDa surface protein in all *Brucella* species and biovars (Ohtsuki et al., 2008). The primers were listed in Table 1 and synthesized by TsingKe Biotech Co., Ltd (Beijing, China). The primer sequences and their positions in the expression site of the *Bscp31* gene were displayed in Figure 1.

**Table 1:** The primers used in the current report.

**Primers^**a**^**	**Sequences and modifications^**b**^**	**Length^**c**^**	**Genes**
F1	5′-TCGGTTGCCAATATCAATGC-3′	20 nt	*Bscp31*
F2	5′-GAACCCGGCTCATCCAG-3′	17 nt	
CP1	5′-CGTTATAGGCCCAATAGGCAACGTATCAAGTCGGGCGCTCT-3′	41 mer	
CP2	5′-GGCGACGCTTTACCCGGAAAGCCTTTCAGGTCTGCGA-3′	37 mer	
C1	5′-CGTTATAGGCCCAATAGGCAACGT-3′	24 nt	
C1^*^	5′-FITC-CGTTATAGGCCCAATAGGCAACGT-3′	24 nt	
C2	5′-GGCGACGCTTTACCCGGAAA-3′	20 nt	
D1	5′-ACTGCGTAAAGCCGGACT-3′	18 nt	
D2	5′-CGTAAGGATGCAAACATCAA-3′	20 nt	
R1	5′-GCCCTTGCCATCATAAAG-3′	18 nt	
R2	5′-AAGGTGGAAGATTTGCG-3′	17 nt	

a C1^*^, 5′-labeled with FITC when used in MCDA-LFB assay.

b *FITC, fluorescein isothiocyanate*.

c *mer, monomeric unit; nt, nucleotide*.

### Bacterial Strains and Genomic DNA Preparation

A total of 97 bacterial strains, including *B. melitensis, B. abortus, B. canis, B. ovis, B. suis, B. neotomae*, and 18 non-*Brucella* strains were used in this study (Table 2). The vaccine strain of *B. suis* (GZ-CDC-S2) was selected for the optimization of target method. The genomic templates were extracted using DNA extraction Kites (QIAamp DNA minikits, Hilden, Germany) according the manufacturer's instructions. Then, the templates were quantified using ultraviolet spectrophotometer (Nano drop ND-1000, Calibre, Beijing, China) at A260/280.

**Table 2:** Bacterial strains used in the current study.

**Bacteria**	**Strain no. (source of strains)^**a**^**	**No. of strains**	**MCDA-LFB result^**b**^**
**BRUCELLA SPECIES**			
*B. suis*	Vaccine strain (GZ-CDC-S2)	1	P
*B. suis*	Isolated strains	1	P
*B. melitensis*	Vaccine strain (GZ-CDC-M5)	1	P
*B. melitensis*	Isolated strains	69	P
*B. abortus*	Vaccine strain (GZ-CDC-A19)	1	P
*B. abortus*	Isolated strains	3	P
*B. canis*	Isolated strains	1	P
*B. ovi*	Isolated strains	1	P
*B. neotoma*	Isolated strains	1	P
**NON-BRUCELLA SPECIES**			
*Salmonella*	Isolated strains	1	N
*Bacillus cereus*	Isolated strains	1	N
*Shigella dysenteriae*	Isolated strains	1	N
*Shigella boydii*	Isolated strains	1	N
*Shigella flexneria*	Isolated strains	1	N
*Shigella sonneri*	Isolated strains	1	N
*Listeria monocytogenes*	ATCC-EGD-e	1	N
*Enterococcus faecalis*	ATCC35667	1	N
*Enterococcus faecium*	Isolated strains	1	N
*Pseudomonas aeruginosa*	Isolated strains	1	N
*Streptococcus pneumonia*	ATCC700674	1	N
*Klebsiella pneumoniae*	Isolated strains	1	N
*Vibrio cholera*	Isolated strains	1	N
*Enterotoxigenic E. coli*	Isolated strains	1	N
*Neisseria.meningitidis*	Isolated strains	1	N
*Bordetella pertussis*	Isolated strains	1	N
*Staphylococcus aureus*	Isolated strains	1	N
*Leptospira interrogans*	Isolated strains	1	N

a *ATCC, American Type Culture Collection; GZ-CDC, Guizhou Center for Disease Control and Prevention*.

b *P, positive; N, negative. Only Brucella species strains could be detected by the MCDA-LFB technique, indicating the extremely high selectivity of the method*.

### Preparation and Operation of Lateral Flow Biosensor (LFB)

Lateral flow biosensor (LFB) was constructed according to previous reports and used for indicating the MCDA results (Wang et al., 2017). Briefly, the biosensor consists of a backing pad, an immersion pad, a conjugate pad, NC membrane, and an absorbent pad. SA-PNPs (Dye streptavidin coated polymer nanoparticles) were gathered in the conjugate pad. Anti-FITC and biotin-BSA were affixed at the test line (TL) and control line (CL), respectively.

### MCDA Reactions

*Brucella*-MCDA reactions were conducted in a one-step reaction in a 25-μl mixture containing 2.5 μl 10 X of the supplied buffer, 0.4 μM each of displacement primers F1 and F2, 0.8 μM each of amplification primers C1^*^, C2, R1, R2, D1, and D2, 1.6 μM each of cross primers CP1 and CP2, 0.8 M betaine (Sigma-Aldrich), 1.4 mM dATP, 1.0 mM dCTP, 0.4 mM biotin-14-dCTP, 1.4 mM dGTP, 1.4 mM dUTP, 1 μl (8 U) of *Bst* 2.0 polymerase, 0.3 μl (0.3 U) of AUDG, and 1 μl DNA template. A total of three detection methods, including real-time turbidity (LA-320C), colorimetric indicator (Malachite Green, MG) and LFB detection, are employed for the confirming and verifying the *Brucella*-MCDA products. The strategy of visualizing amplification products on LFB was adapted from previous publications (Wang et al., 2018b).

We then examined the optimal temperatures of *Brucella*-MCDA primers during the amplification stage. Temperatures ranging from 60 to 67°C (with 1°C intervals) were tested and compared. MCDA mixtures with 1 μl of DNA template of *Salmonella* (isolated strain) and *Bacillus cereus* (isolated strain) were employed for negative controls (NC), and 1 μl of double distilled water (DW) were employed as a blank control (BC).

### Analytical Sensitivity of MCDA-LFB Assay

In this report, we determined the analytical sensitivity of MCDA-LFB method using the serial dilution of *B. suis* (GZ-CDC-S2), which cover the range of 10 ng μl^−1^ to 100 ag μl^−1^ (10 ng, 10 pg, 1 pg, 100 fg, 10 fg, 1 fg, and 100 ag per microliter). A volume of 1 μl of genomic template was added into the MCDA reaction mixtures. As described above, MCDA reactions were performed at a constant temperature to determine the limit of detection (LoD), which was confirmed as the last dilution of each positive test.

Particularly, optimal duration of time required for MCDA-LFB approach during the isothermal amplification stage also was examined. A total of four reaction times, including 30, 40, 50, and 60 min, were tested and compared at the optimal reaction condition, and the amplification results were indicated using LFB.

### Simulating Carryover Contamination

The MCDA amplicons produced from 10 pg/μl in absence of AUDG enzyme were quantitated using ultraviolet spectrophotometer (NanoDrop ND-1000, Calibre, Beijing, China), which were used for making serial dilution from 1 × 10^−13^ to 1 × 10^−20^ g μL^−1^. A volume of 1 μl of each dilution was employed as the templates for MCDA reactions, which were used as the source of simulating carryover contaminants.

### Prevention of Carryover Contamination by AUDG Enzyme

To demonstrate the usability of AUDG enzyme to remove the unwanted results due to carryover contamination, MCDA reactions with AUDG and without AUDG were conducted and compared by adding 1 μl of diluted templates (10 ng, 10 pg, 1 pg, 100 fg, 10 fg, 1 fg, and 100 ag per microliter) and 1 μl of simulated carryover contamination of 1 × 10^−18^ g μl^−1^ in the same reaction tube. For each MCDA reaction, total mass of simulated carryover contaminants (1 × 10^−18^ g) is approximately equivalent to a 0.2 μm-diameter aerosol droplet, which did not be completely prevented by either high efficiency particulate air filters or fibrous pipette tip filters in the biosafety cabinets (Le Rouzic, 2006; Barhate and Ramakrishna, 2007; Wang et al., 2018a). The LoD of MCDA method without AUDG and with AUDG cleavage before isothermal reaction was compared to verify whether AUDG enzyme is capable of eliminating undesired results in the MCDA assays.

### Analytical Specificity of MCDA-LFB Assay

In the current report, we examined the analytical specificity of MCDA-LFB using DNA templates (at least 10 ng per microliters) from 79-*Brucella* strains to 18 non-*Brucella* strains (Table 2). All MCDA results were reported using lateral flow biosensor and all tests were repeated three times.

### Examination of the Feasibility of MCDA Assay

A total number of 156 whole blood samples of human and goat suspected from Brucellosis were obtained from distinct regions of Guizhou province, China. All the 156 samples were subjected for *Brucella* spp. detection using traditional culture, PCR, and MCDA-LFB methods. Around 3 mL of venous blood collected from the goat and human was aseptically injected into a two-phase culture flask (BIOVD, Zhengzhou, Henan, China) for cultivation. After incubation at 37°C and 5% CO_2_ atmosphere for 3–5 days (blind passage for more 3–5 days of cultivation may be needed), the *Brucella* suspicious bacteria strain was streaked on both a blood agar plate and a *Brucella* agar plate for further identification. Traditional biological methods including colony morphology, Gram stain, CO_2_ requirements, H_2_S production, agglutination with monospecific antisera, and phage lysis test, were used for the identification of the *Brucella* suspicious bacterial isolates (Yang et al., 2018). Simultaneously, genomic templates from the 156 whole blood samples were extracted using protocol of QIAamp. PCR performance of all the 156 DNA templates was conducted using *Brucella* spp. specific primers (B4 and B5 primers) targeting *bscp31* gene having an amplicon size of 224 bp (Baily et al., 1992). The results obtained from MCDA-LFB assay were compared with conventional culture and PCR test.

## Results

### Demonstration and Detection of MCDA Products

**Figure 2:**
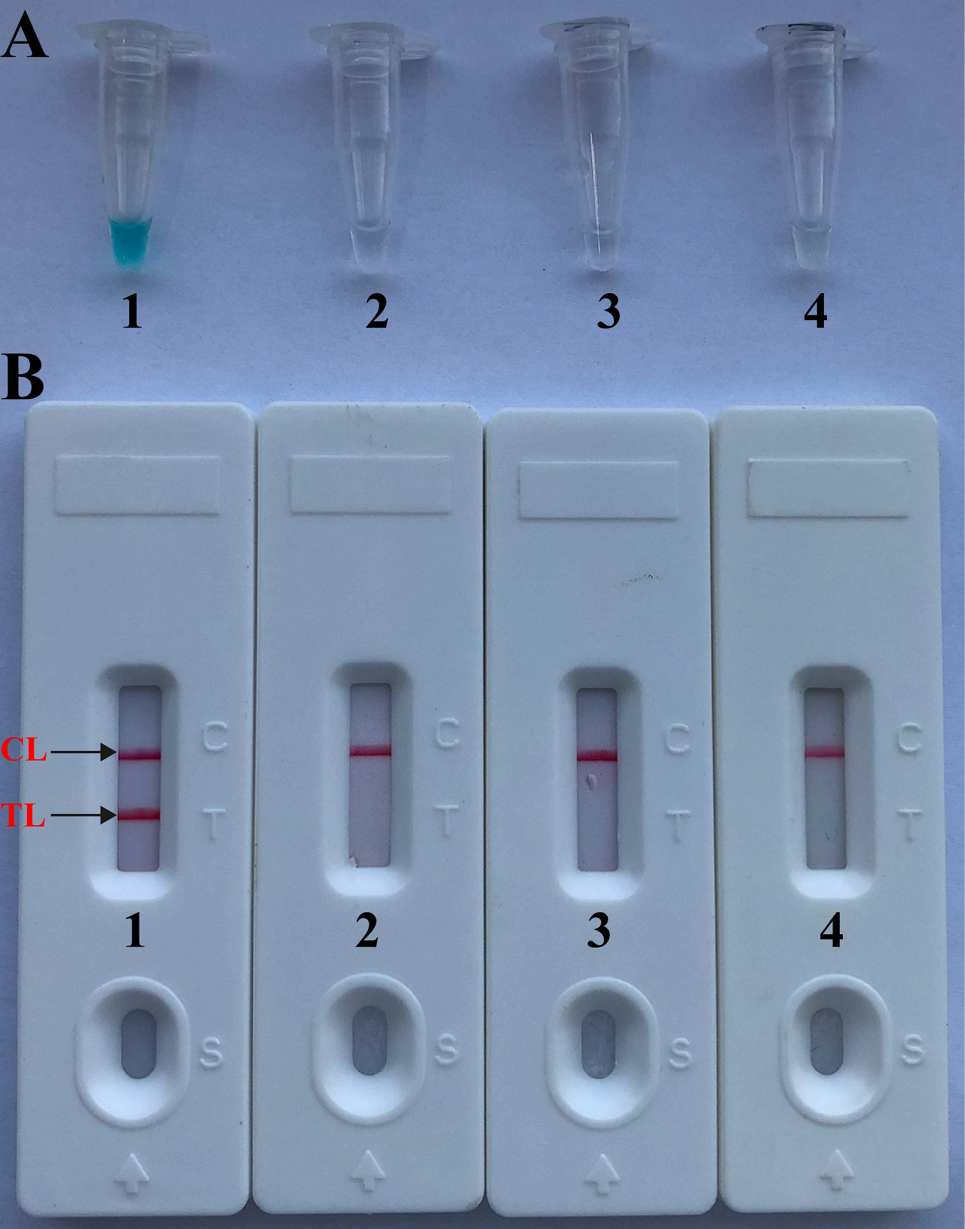
Confirmation and verification of *Brucella*-MCDA products. **(A)** Color change of *Brucella*-MCDA tubes; **(B)** LFB applied for visual detection of *Brucella*-MCDA products. Tube A1 (biosensor B1), positive amplification; tube A2 (biosensor B2), negative amplification (*Salmonella*), tube A3 (biosensor B3), negative amplification (*Bacillus cereus*), tube A4 (biosensor B4), negative control (DW).

As shown in Figure 2, the color change from blue to light green when the samples are positive is visible to the unaided eye under natural light. However, the color change from blue to colorlessness when the samples are negative is visible to the naked eye under natural light (Figure 2A).

By LFB, a volume of 1 μl of MCDA products (FITC and biotin-labeled MCDA amplicons) was firstly loaded into the immersion pad. Then, a 60 μl aliquot of running buffer also was loaded into the immersion pad. The capillary flow is able to transfer amplicons and SA-PNPs from the conjugate pad to TL (Test line) and CL (Control line). Biotin-labeled products form complex with SA-PNPs via biotin-streptavidin-biotin interactions at the conjugated pad, and biotin/MCDA complexes were captured at the TL by interaction between anti-FITC and FITC. In particular, SA-PNPs that did not form complexes were immobilized at the CL by interaction between biotin and streptavidin. As a result, FITC/MCDA/SA-DNPs complexes and non-complexed SA-PNPs were indicated by crimson red lines at the TL and CL, respectively. Two crimson red lines (TL and CL) were seen for in positive results, and only the CL appeared in the negative and blank control (Figure 2B). Herein, the primer set designed in the report was available for developing MCDA-based assays for rapid detection of *Brucella* spp. strains.

### Optimal Reaction Temperature of MCDA Primer Set

**Figure 3:**
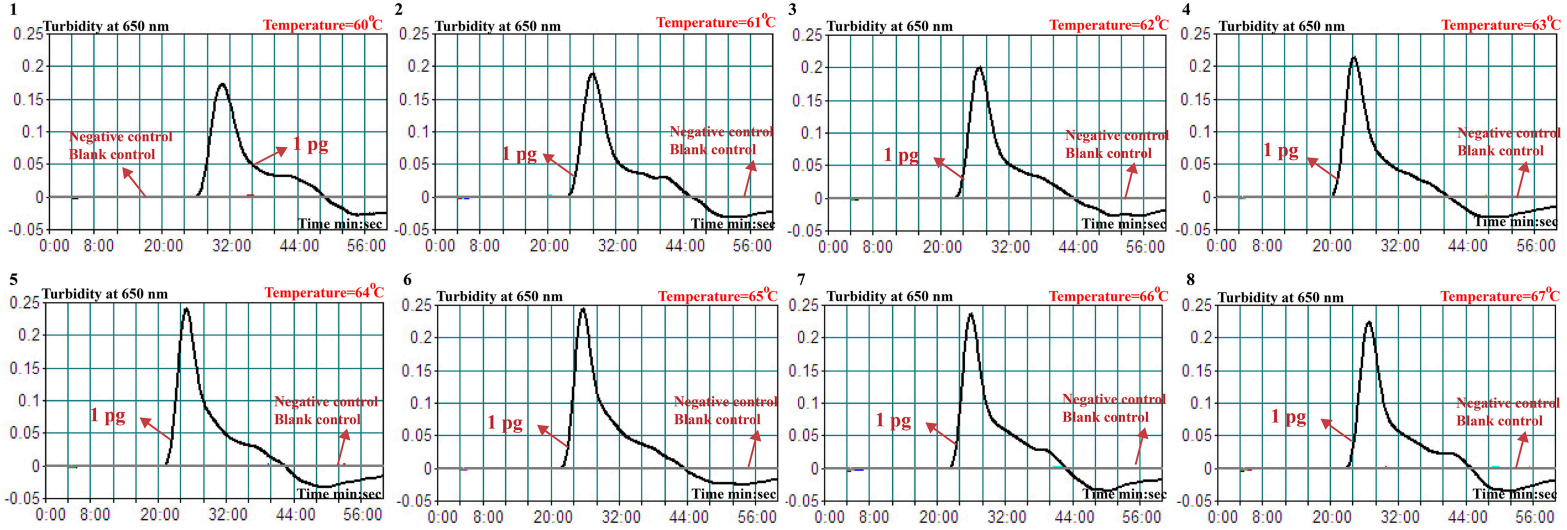
Optimal temperature for *Brucella*-MCDA primer set. The MCDA reactions for detection of *Bscp31* gene were monitored by real-time measurement of turbidity and the corresponding curves of concentrations of templates were marked in the figures. The threshold value was 0.1 and the turbidity of >0.1 was considered to be positive. Eight kinetic graphs (1–8) were obtained at various temperatures (60–67°C, 1°C intervals) with target pathogens DNA at the level of 1 pg per tube. The graphs from 3 (62°C) to 6 (65°C) showed robust amplification.

A total of eight temperatures (60–67°C, with 1°C interval) were compared under standard MCDA protocol described above. Real-time turbidity was employed for monitoring the *Brucella*-MCDA reactions, and all examined temperatures yielded the kinetics graphs (Figure 3). Faster amplifications were observed for assay temperature of 62–65°C. Amplification temperature of 63°C was selected as optimal amplification condition, and was used for performing the rest of MCDA reactions conducted in the current report.

### Analytical Sensitivity of *Brucella*-MCDA Assay

**Figure 4:**
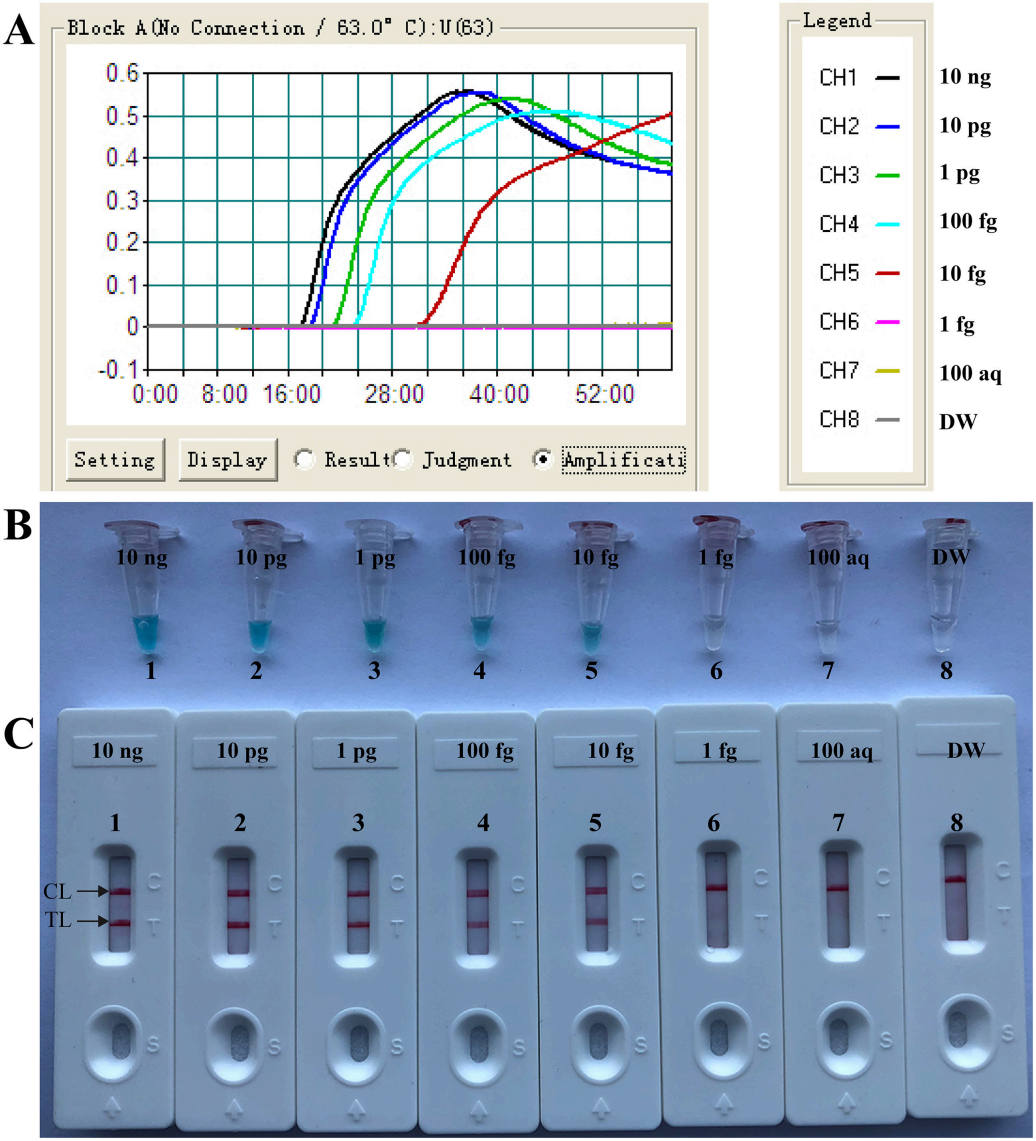
, Sensitivity of MCDA-LFB assay using serially diluted genomic templates with *B. suis* strain GZ-CDC-S2. Signals **(A)**/Tubes **(B)**/Biosensors **(C)** 1–8 represented the DNA levels of 10 ng, 10 pg, 1 pg, 100 fg, 10 fg, 1 fg, 100 atto gram per reaction and blank control (DW). The genomic DNA levels of 10 ng to 10 fg per reaction produced the positive reactions.

As showing in Figure 4, *Brucella*-MCDA assay was able to detect down to 10 fg genomic DNA per vessel. Two crimson bands (TL and CL) simultaneously appeared on the biosensors, indicating the positive results for *Bscp31* gene (Figure 4C). The sensitivity of *Brucella*-MCDA obtained from biosensor was in complete accordance with real-time turbidity analysis (Figure 4A) and colorimetric indicator detection (Figure 4B).

### *Brucella*-MCDA Detect Simulated Carryover Contamination

**Figure 5:**
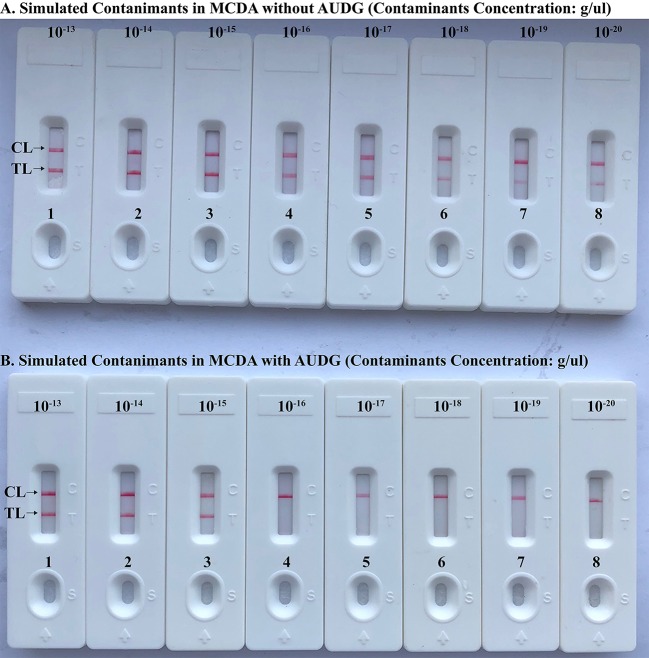
Control of carryover contamination in MCDA-LFB assay. Sensitivity examination of MCDA without AUDG treatment **(A)** and MCDA with AUDG treatment **(B)** using 10-fold serial dilutions of simulated carryover contamination (dUTP-incorporated products, concentration diluted from 1 × 10^−13^, 1 × 10^−14^, 1 × 10^−15^, 1 × 10^−16^, 1 × 10^−17^, 1 × 10^−18^ 1 × 10^−19^, and 1 × 10^−20^ g μL^−1^) as determined using LFB.

To verify that the amplification products from *Brucella*-MCDA method are sufficient to contaminate new *Brucella*-MCDA reaction, the serial dilution of *Brucella*-MCDA amplicons (1 × 10^−13^, 1 × 10^−14^, 1 × 10^−15^, 1 × 10^−16^, 1 × 10^−17^, 1 × 10^−18^, 1 × 10^−19^, and 1 × 10^−20^ g/μL) was prepared for performing *Brucella*-MCDA amplifications. In *Brucella*-MCDA assay without AUDG digestion, MCDA was able to detect down to 1 × 10^−20^ g of simulated carryover contaminant per reaction (Figure 5A). In parallel, *Brucella*-MCDA amplification with AUDG digestion can only detect 1 × 10^−15^ g of simulated carryover contaminant per tube (Figure 5B). Our data demonstrated that a source contaminant (1 × 10^−18^ g/μL), which is equivalent to a 0.2 μm-diameter aerosol droplet and can't be efficiently eliminated by fibrous pipette tip filters, is sufficient to contaminate new *Brucella*-MCDA amplifications. In addition, *Brucella*-MCDA assay with AUDG enzyme can prevent the reactions of up to 10^5^-fold higher concentration of carryover contaminant amplicons, which significantly decrease the likelihood of unwanted results in *Brucella*-MCDA detection.

### *Brucella*-MCDA Method With AUDG Enzyme Removes False-Positive Amplifications

**Figure 6:**
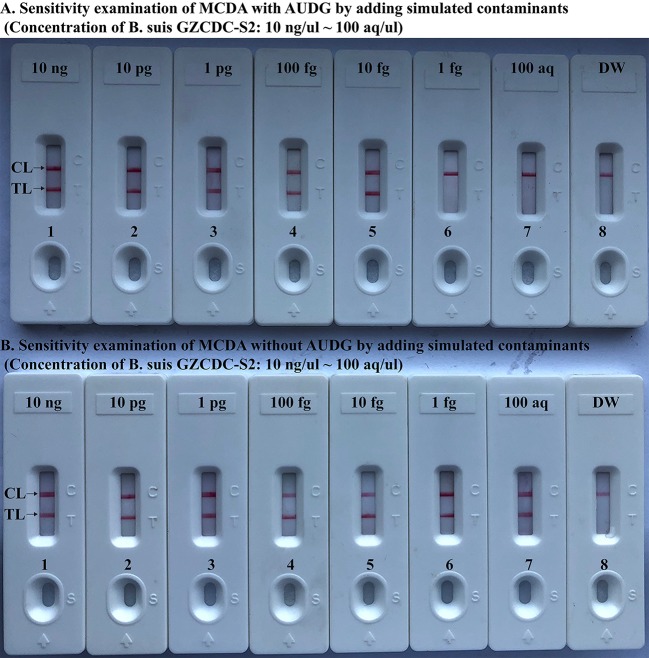
MCDA-LFB assay eliminates false-positive detection due to carryover contamination. Sensitivity test of MCDA-LFB assay with AUDG treatment **(A)** and MCDA-LFB assay without AUDG treatment **(B)** using serial dilutions (10 ng μl^−1^, 10 pg μl^−1^, 1 pg μl^−1^, 100 fg μl^−1^, 10 fg μl^−1^, 1 fg μl^−1^, and 100 ag μl^−1^) of GZ-CDC-S2 and 1 × 10^−18^ g μL^−1^ of simulated carryover contamination (dUTP-incorporated m-MCDA products) as determined using LFB.

To further validate that *Brucella*-MCDA assay using AUDG enzyme has the ability to reduce the likelihood of undesired results due to carryover contamination. Sensitivity evaluation of *Brucella*-MCDA without AUDG and AUDG enzyme is conducted using serial dilution of *B. suis* GZ-CDC-S2 templates, and contaminants (dUTP-incorporated amplicons) at the level of 1 × 10^−18^ g μl^−1^ also is added into reaction tube. In *Brucella*-MCDA reactions with AUDG digestion, assay's sensitivity was consistent with the aforementioned sensitivity test (Figures 4, 6A). Comparing with *Brucella*-MCDA without AUDG cleavage, all tested samples exhibited positive amplifications, even including examined samples with undetectable level of *B. suis* GZ-CDC-S2 templates (<1 fg per reaction), which are regarded as false-positive results (Figure 6B). Therefore, we can't correctly examine the sensitivity of *Brucella*-MCDA without AUDG treatment. These data indicated that *Brucella*-MCDA method developed here could successfully remove the false-positive results generating from carryover contamination.

### The Time Optimization of the *Brucella*-MCDA Assay

**Figure 7:**
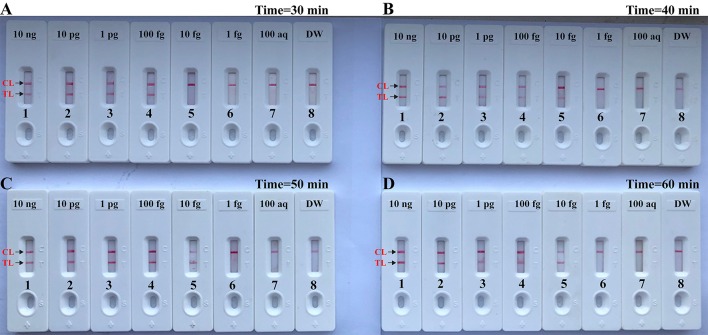
Optimal duration of time required for MCDA-LFB assay. Four different reaction times [**(A)**, 30 min; **(B)**, 40 min; **(C)**, 50 min; and **(D)**, 60 min] were tested and compared at 63°C. Biosensors 1, 2, 3, 4, 5, 6, 7, and 8 represent DNA levels of 10 ng μl^−1^, 10 pg, 1 pg μl^−1^, 100 fg, 10 fg μl^−1^, 1 fg μl^−1^, 100 ag μl^−1^, and blank control (DW). The best sensitivity was seen when the amplification lasted for 40 min **(B)**.

In this report, we also examined the optimum time for *Brucella*-MCDA method during the reaction stage. A total of four amplification times, ranging from 30 to 60 min (at 10 min interval), were examined and compared at optimal amplification temperature (63°C). The lowest level of DNA templates (10 fg) was detected when *Brucella*-MCDA amplification only lasted 40 min at 63°C (Figure 7). Hence, an isothermal time of 40 min was employed as the optimal MCDA reaction time for the rest MCDA performances conducted in the current report. The whole process, including template preparation (20 min), AUDG digestion (5 min), *Brucella*-MCDA reaction (40 min), and result reporting (2 min), could be completed within 70 min.

### Specificity of *Brucella*-MCDA-LFB Assay

**Figure 8:**
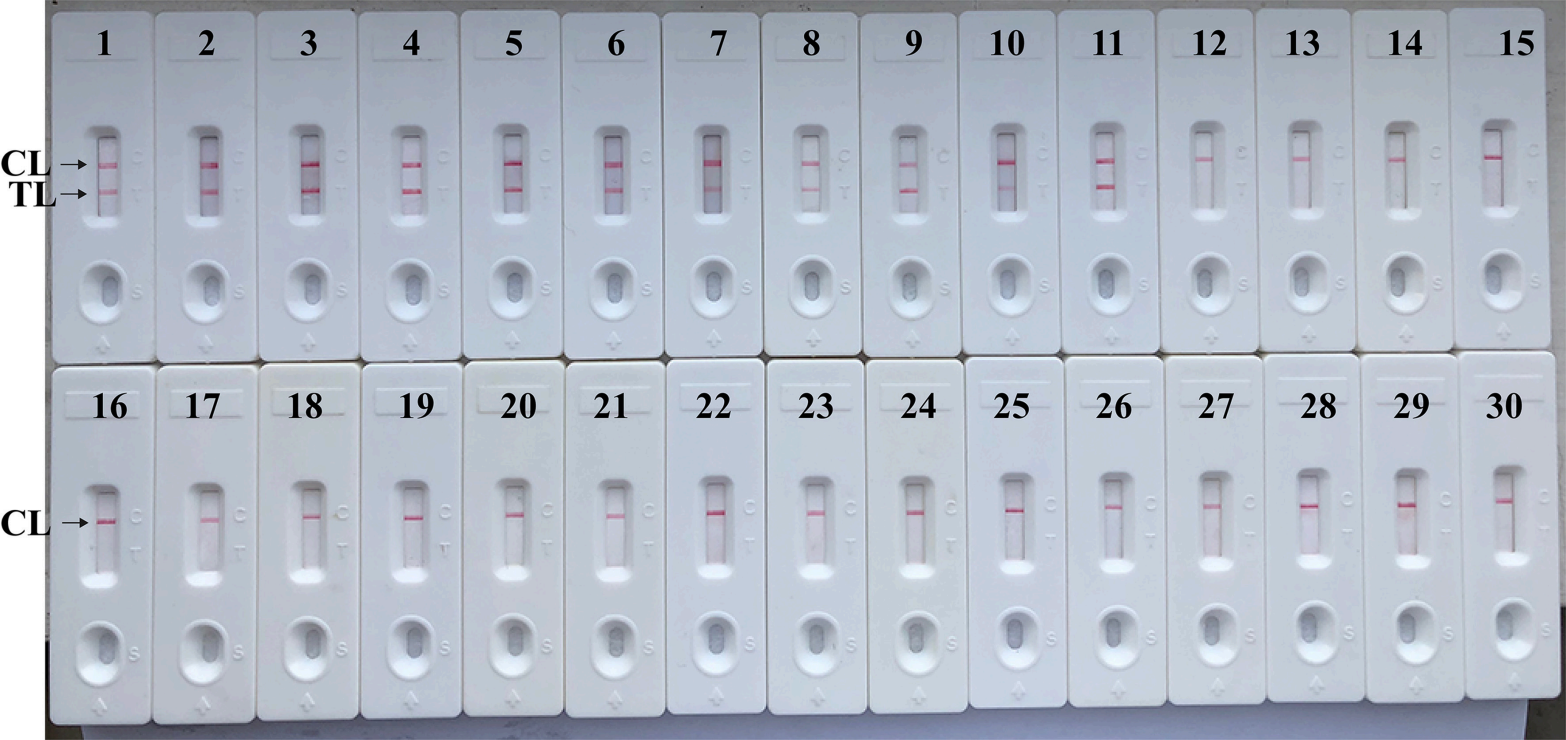
Analytical Specificity of MCDA-LFB assay using different bacterial strains. The MCDA-LFB was evaluated using different genomic DNA templates. Biosensor 1, *B. suis* (GZ-CDC-S2); biosensor 2, *B. melitensis* (GZ-CDC-M5); biosensor 3, *B. abortus* (GZ-CDC-A19); biosensor 4, *B. suis* (isolated strain); biosensor 5–8, *B. melitensis* (isolated strains); biosensor 9, *B. abortus* (isolated strain); biosensor 9, *B. canis* (isolated strain); biosensor 10, *B. ovi* (isolated strain); biosensor 11, *B. neotoma* (isolated strain); Biosensor 12–29, *Salmonella*; *Bacillus cereus*; *Shigella dysenteriae*; *Shigella boydii*; *Shigella flexneria*; *Shigella sonneri*; *Listeria monocytogenes*; *Enterococcus faecalis*; *Enterococcus faecium*; *Pseudomonas aeruginosa*; *Streptococcus pneumonia*; *Klebsiella pneumoniae*; *Vibrio cholera*; *Enterotoxigenic E. coli*; *Neisseria meningitidis*; *Bordetella pertussis*; *Staphylococcus aureus*; *Leptospira interrogans*; Biosensor 30, negative control (DW).

The assay's specificity is evaluated using DNA templates from *Brucella* strains and non-*Brucella* strains. After a 40 min reaction at the optimal conditions, only the templates extracted from *Brucella* strains produced the positive results (Figure 8 and Table 2). Test line (TL) and control line (CL) simultaneously appeared on biosensor, suggesting the positive results for target pathogens (Figure 8, biosensor 1–11). Only control line (CL) appeared on the biosensor, indicating the negative results for non-*Brucella* strains and blank control (Double distilled water, DW). Our data verified that MCDA-LFB assay established in the current report is able to correctly identify all target pathogens (Figure 8 and Table 2). No cross-reactions to non-*Brucella* strains were observed according to the analytical specificity determination. These results demonstrated that the MCDA-LFB assay displayed high selectivity for detection of *Brucella* strains.

### Evaluation of the MCDA-LFB Using Whole Blood Sample of Human and Goat

In order to further examine the suitability of MCDA-LFB as a useful tool for *Brucella* detection, a total of 156 whole blood samples of human and goat suspected from Brucellosis were examined using culture-biotechnical method, conventional PCR and MCDA-LFB assays. Genomic templates were directly extracted from the 156 whole blood samples, which were used for conducting *Brucella*-MCDA-LFB and *Brucella*-PCR. The results were summarized in Table 3. In the case of whole blood samples, 17 (10.99%), and 14 (8.97%) whole blood samples were *Brucella*-positive by MCDA-LFB and conventional PCR, respectively (Table 3). The MCDA-LFB detection accuracy obtained was 100% when compared to the conventional culture-biotechnical technique (10.99%). Our data exhibited that the *Brucella*-MCDA-LFB method developed in this study was a valuable tool for *Brucella* detection, and had higher detection ability when compared to PCR diagnostic.

**Table 3:** Comparison of conventional PCR, culture-biotechnical, and MCDA-LFB assays for the detection of *Brucella* species in whole blood samples of human and goat.

**Detection methods**	**Whole blood samples (*****n*** **= 156)**	
	**Positive**	**Negative**
PCR	14	142
Culture	17	139
MCDA-LFB	17	139

## Discussion

*Brucella spp*. is capable of causing disease in both animals and human, and human brucellosis, regarding as a life-threating disease, is the results of animal products consumption, direct contact with infected animals or animal carcass (Irvem et al., 2015). There is an urgent requirement to devise a method for reliable detection of *Brucella* with acceptable simplicity, rapidity, sensitivity, and specificity, especially for field, basic, and clinical laboratories. To achieve more such effective diagnostic tool, a MCDA-LFB method targeting *Bscp31* gene was successfully established for detecting *Brucella* spp. strains. A set of MCDA primer, which specially recognized 10 regions of the *Bscp31* gene, was designed on the basis of MCDA principle, thus provided a high degree of specificity for *Brucella* spp. detection (Figure 1). To confirm the analytical specificity of MCDA-LFB assay, genomic templates extracted from 79 *Brucella* strains to 18 non-*Brucella* strains were successfully examined (Figure 8 and Table 2). Positive results were obtained only from the assay of all *Brucella* strains but not for non-*Brucella* strains. These data suggested that MCDA-LFB assay targeting the *Bscp31* gene identified *Brucella* spp. strains with 100% specificity.

In this report, we employed the lateral flow biosensor (LFB) to detect reaction products, because of its rapid results, simple operation, and ease of use in field, basic, and clinical settings. Comparing with the other measurement methods (i.e., colorimetric indicator and turbidity) employed in the current report (Figures 2, 3), detection of MCDA products using biosensor was not only simpler, but also more rapid and less error-prone. Particularly, analysis of MCDA products using biosensor could eliminate the use of special instrument, reagent, and additional procedures, thus LFB was more suitable than other detection techniques for simple, visual, and rapid indication of amplification results.

To obtain the visual and objective analysis of amplification products using biosensor, opening of the MCDA reaction tube is an essential step. As a result, aerosol droplets of different sizes, which contain high concentration of amplicons, are yielded in this step. Hence, false-positive result arising from previous MCDA reactions is one of very tricky problems, because MCDA technique is extremely vulnerable to contaminants (Wang et al., 2018a). Our data indicated that a trace amount of amplicons (1 × 10^−20^ g/vessel) is able to generate the unwanted results, thus preventing the carryover contamination is a key factor for reliable diagnosis. The current report successfully eliminated the carryover contamination using two additional components, including dUTP and AUDG enzyme. In the MCDA system, dUTP was incorporated instead of dTTP into all reaction products. Prior to the start of next round of amplification, reaction mixtures were treated with AUDG enzyme at the room temperature for only 5 min, and the products from previous amplification can be specifically cleaved by the AUDG enzyme (Wang et al., 2017). The natural nucleic acid templates remain completely unaffected, because they are uracil-free DNA (Kil et al., 2015). Importantly, the AUDG, as a heat-labile enzyme, is rapidly and automatically deactivated when MCDA amplification is conducted at an elevated temperature (i.e., 63°C). Genuine products subsequently yielded from the target DNA could not be digested, permitting MCDA reaction to normally proceed. Thus, the use of heat-labile enzyme enables the MCDA technique to conducted in a single closed tube (Wang et al., 2017). The whole procedure, including template preparation (20 min), AUDG digestion (5 min), isothermal reaction (40 min), and result reporting (2 min), was finished within 80 min.

In this report, the *Brucella*-MCDA-LFB method developed here for detecting *Bscp31* gene was 10 fg of nucleic acid templates per vessel, and the analytical sensitivity using LFB analysis of MCDA products was in complete accordance with colorimetric indicator (MG) analysis and real-time turbidity detection (Figure 4). Then, to further determine the practical feasibility of MCDA-LFB detection to *Brucella* spp., we examined 156 whole blood samples of human using MCDA-LFB detection, PCR analysis, culture bio-technique method. The MCDA-LFB assay showed high analysis sensitivity for target pathogens compared to PCR technique. Three blood samples were demonstrated to be positive by MCDA-LFB assay and culture-biotechnical approach, but negative by conventional PCR diagnostic. The lower detection rate of PCR assay may be due the reasons that the copy numbers of *Brucella* spp. nucleic acids were lower than the analytical sensitivity or the presence of some inhibitors specific to the PCR method affected the diagnostic sensitivity. Comparing with PCR and culture-based techniques, MCDA-LFB assay can be performed with only simple equipment (such as a regular laboratory bath or heat block) that offers a fixed temperature of 63°C, eliminating the long turnaround times and avoiding the use of complex instrument.

In summary, a MCDA-LFB method for detection of *Brucella* spp. strains based on *Bscp31* gene were successfully developed and evaluated. *Brucella*-MCDA-LFB assay displayed high specificity for target pathogen detection, and had the analytical sensitivity of 10 fg per reaction with pure culture. By biosensor, the amplification products were visually analyzed, which was easy-to-use, disposable and objective. Most important, the unwanted amplifications arising from carryover contamination could be removed by using dUTP and AUDG enzyme. Hence, *Brucella*-MCDA-LFB developed here was a rapid, simple, reliable and sensitive technique to identify all *Brucella* spp. strains, and could be used as potential screening tool for *Brucella* strains in field, basic, and clinical laboratory.

## Author Contributions

YiW and SL conceived and designed the experiments. SL, YL, YuW, MW, and CL performed the experiments. YiW and SL analyzed the data. YiW, SL, YL, YuW, MW, and CL contributed the reagents, materials, analysis tools. YiW performed the software. YiW and SL wrote the paper.

### Conflict of Interest Statement

The authors declare that the research was conducted in the absence of any commercial or financial relationships that could be construed as a potential conflict of interest.
